# Spatial Memory and Long-Term Object Recognition Are Impaired by Circadian Arrhythmia and Restored by the GABA_A_Antagonist Pentylenetetrazole

**DOI:** 10.1371/journal.pone.0072433

**Published:** 2013-08-29

**Authors:** Norman F. Ruby, Fabian Fernandez, Alex Garrett, Jessy Klima, Pei Zhang, Robert Sapolsky, H. Craig Heller

**Affiliations:** 1 Biology Department, Stanford University, Stanford, California,United States of America; 2 Department of Psychiatry and Behavioral Sciences, Stanford University, Palo Alto, California, United States of America; Kent State University, United States of America

## Abstract

Performance on many memory tests varies across the day and is severely impaired by disruptions in circadian timing. We developed a noninvasive method to permanently eliminate circadian rhythms in Siberian hamsters (*Phodopussungorus*) so that we could investigate the contribution of the circadian system to learning and memory in animals that are neurologically and genetically intact. Male and female adult hamsters were rendered arrhythmic by a disruptive phase shift protocol that eliminates cycling of clock genes within the suprachiasmatic nucleus (SCN), but preserves sleep architecture. These arrhythmic animals have deficits in spatial working memory and in long-term object recognition memory. In a T-maze, rhythmic control hamsters exhibited spontaneous alternation behavior late in the day and at night, but made random arm choices early in the day. By contrast, arrhythmic animals made only random arm choices at all time points. Control animals readily discriminated novel objects from familiar ones, whereas arrhythmic hamsters could not. Since the SCN is primarily a GABAergic nucleus, we hypothesized that an arrhythmic SCN could interfere with memory by increasing inhibition in hippocampal circuits. To evaluate this possibility, we administered the GABA_A_ antagonist pentylenetetrazole (PTZ; 0.3 or 1.0 mg/kg/day) to arrhythmic hamsters for 10 days, which is a regimen previously shown to produce long-term improvements in hippocampal physiology and behavior in Ts65Dn (Down syndrome) mice. PTZ restored long-term object recognition and spatial working memory for at least 30 days after drug treatment without restoring circadian rhythms. PTZ did not augment memory in control (entrained) animals, but did increase their activity during the memory tests. Our findings support the hypothesis that circadian arrhythmia impairs declarative memory by increasing the relative influence of GABAergic inhibition in the hippocampus.

## Introduction

The use of novel environments to stimulate exploratory behavior has proven useful for evaluating mechanisms of recognition and spatial memory. Spatial working memory, for example, can be evaluated in a T-maze where alternating goal arm choices are made even in the absence of any reinforcement and is thus considered to be spontaneous [1]. Spontaneous alternation (SA) behavior is a nearly ubiquitous phenomenon, having been demonstrated in a wide range of vertebrate and invertebrate species [2]. The popularity of the SA test has risen in recent years due to its simplicity of use and sensitivity in revealing damage to the septal-hippocampal system [3]–[8]. Novel object recognition (NOR) also takes advantage of the innate tendency to explore novel objects and environments [9]–[11]. This test relies primarily on the perirhinal-entorhinal cortices, but also depends on the hippocampus when tests are conducted in an open arena requiring spatial exploration [12]–[14].

We have been investigating how the circadian system modulates SA and NOR because circadian timing that is disrupted either by living on non-24 h schedules or by repeatedly phase-shifting the lighting cycle impairs memory in humans and rodents [15]–[21]. Eliminating circadian rhythms should reveal circadian contributions to memory, but current methods for inducing arrhythmia are problematic for studies of cognition. Surgical ablation of the suprachiasmatic nucleus (SCN) and constant light exposure can increase stress hormone levels [22]–[25], and lesions damage areas adjacent to the SCN [26]–[28]. Clock gene knockouts can cause arrhythmia, but impair sleep regulation which can indirectly interfere with learning and memory [25], [29]–[31].

Siberian hamsters (*Phodopussungorus*) are well suited for functional studies of the circadian system because a phase-advancing and phase-delaying light signal administered on two successive nights can permanently eliminate their circadian rhythms in behavior and in clock gene expression in the SCN, while leaving animals neurologically and genetically intact [32]–[34]. Circadian arrhythmia induced in this manner has no effect on sleep architecture or on homeostatic responses to sleep deprivation, which allows us to study the impact of arrhythmia on memory without the confound of altered sleep patterns [35].

Our previous work demonstrated that arrhythmic hamsters had substantial deficits in short-term object recognition memory that could be rescued by the GABA_A_ antagonist pentylenetetrazole (PTZ) [36]. GABA is the primary neurotransmitter of the SCN [37]–[38], and SCN GABA levels oscillate with a daily rhythm [39], [40]. Because our hamsters have an arrhythmic SCN, we hypothesized that the daily pattern of GABA output from the SCN would likely be disrupted as well, which could potentially result in chronically elevated levels of inhibition to downstream targets. One such target is the lateral/medial septum that provides the primary subcortical cholinergic input to the septo-hippocampal pathway, which is important for declarative memory [36], [41], [42]. In light of these considerations, we evaluated whether circadian arrhythmia impairs long-term object recognition and spatial working memory, and whether such deficits could be mitigated by lowering GABAergic inhibition with PTZ. We also evaluated whether PTZ could augment cognition in entrained (rhythmic) hamsters.

## Methods

### Animals and Housing Conditions

Siberian hamsters (*Phodopussungorus*) were bred in the laboratory in a 16:8-h light-dark (LD) cycle (lights on at 0200 h, PST) at an ambient temperature of 22°C. Male and female hamsters were housed two to four per cage in the colony room and then individually in white polypropylene cages (30×17×17 cm; Nalgene) just prior to the beginning of the study. During experiments, hamsters were maintained in six recording chambers (10 individually caged animals per chamber). Each chamber was equipped with its own light source and a photosensor that allowed illumination cycles to be recorded by computer. Animals were provided with cotton batting for nesting material; food (Purina chow # 5015) and tap water were available *ad libitum*. All experimental procedures were approved by Stanford University’s Administrative Panel on Laboratory Animal Care (Animal Use Protocol Number: 14988) and were conducted in accordance with the NIH Guide for the Care and Use of Laboratory Animals.

### Lighting Conditions

Light fixtures illuminating both the room where light pulses were administered and the activity recording chambers contained two cool white fluorescent tubes (4,100 K, Philips 40 W) producing an intensity of 10–60 μW/cm^2^ on cage floors when water bottles, food, and cage lids were in place. Variations in light intensity depended on the position of the light meter photocell (International Light Model IL-1405 Radiometer System) within the cage. The light sensor was pointed upward from the cage bottom for these measurements.

### Activity Recording and Analysis

Activity was measured by passive infrared motion detectors mounted directly above the tip of the water bottle sipper tube [33]. In this configuration, activity levels primarily reflected drinking behavior and locomotor activity that occurred directly under the sipper tube. These detectors have a temporal resolution of 1–2 s for successive counts of activity.Activity bouts were summed in 10-min intervals and stored on computer. The times of day when animals were injected or tested is given by zeitgeber time (ZT) where ZT0  =  time of lights-on and ZT16  =  time of lights-off in the animal rooms.

### Spontaneous Alternation in the T-Maze

SA is based on the natural tendency of rodents to consecutively alternate between left and right arm choices during exploration of a T-maze [5], [7], [43]. Hamsters were placed in the start chamber (25 cm×10 cm×20 cm) located at the far end of the stem arm (90 cm) of a transparent acrylic T–maze. A sliding door separated the start chamber from the rest of the apparatus, comprised of an alleyway (65 cm) that leads to a choice point at the intersection of the stem arm with the left and right arms of the maze (each 37 cm×10 cm×20 cm). A divider panel (20 cm×20 cm) is centered at the intersection of the “T” so that it extends 10 cm into the stem arm. The divider panel increases the number of arm entries in a given period of time [7]. Testing was performed under fluorescent lighting (4,100 K, Philips 40 W; 66 μW/cm^2^, 260 lux). Animals spontaneously returned to the start box after each arm entry and rarely “hugged” the divider. Hamsters hugged the divider in <1% of trials and those trials were excluded from the final data set. In preliminary experiments we also tested animals under dim light (<5 lux) during both the light and dark phases of the LD cycle but found no differences in performance (t-test: dim vs. bright light during the day (ZT15), P>0.05; t-test: dim vs. bright light at night (ZT20), P>0.05; data not illustrated).

The protocol for SA testing began with a hamster confined to the start chamber for 60 sec, and then permitted access to the rest of the maze for 7 min. An alternation attempt was scored when all four feet of a hamster entered one of the lateral arms, re–entered the stem arm, and then entered the lateral arm opposite the one previously chosen. Re-entry into the same arm was a nonalternation. Alternation performance was thus operationally defined by the percentage of time the hamster alternated upon arriving at the divider panel (i.e., the number of alternations observed/the number of alternation attempts×100). In studies with mice, the maximum alternation rate typically observed is ∼70% and the minimum is 50%, which represents random chance [7]. To control for odor cues, the T–maze was cleaned with 70% ethanol, dried, and ventilated for a few minutes between trials.

### Object Screening

For the NOR test, four pairs of objects were screened to determine whether the animals exhibited significant preferences for any specific item. Objects were made from various non-porous materials (porcelain, metal, glass, plastic),had various color schemes, and differed in height and shape. A single object of a matched pair was placed along the center of the rear wall of the arena and an individual hamster was allowed to freely explore the object and chamber for 5 min. This was repeated for each of four objects using different groups of hamsters (n = 10 for each object). Each session was videotaped and scored for total exploration time by an independent observer. There were no significant differences among the four objects (mean ± SE exploration time  = 18.0±1.5 sec; one-way ANOVA, P>0.05).

The four objects were then presented in non-matching pairs to determine whether animals exhibited preferences for any one object when two objects were presented together. Six different combinations were tested with different groups of animals (n = 10 for each test). Tests were conducted as described below for the NOR trials. There were no significant differences in exploration times between objects with each trial (t-test, P>0.05), or for the same object across trials when it was matched with the other objects (one-way ANOVA, P>0.05). All objectscreens were performed between ZT11-15.

### Novel Object Recognition

The NOR test is based on the innate tendency of rodents to preferentially explore novel objects over familiar ones [11], [12]. Pairs of objects were chosen at random for each trial. Behavioral testing was carried out in a clear acrylic open-field arena (43×43×31 cm) with a video camera mounted overhead. The arena and camera were enclosed within a cabinet (Med Associates Inc., St. Albans, VT) to minimize noise and visual distractions and to better control lighting. A small ventilation fan provided white background noise. The sample and test phases were performed under very dim light (0.083 μW/cm^2^; 2 lux; measured at the center of the arena floor). Previous studies under bright light produced similar test results, but decreased exploratory activity in the arena [36]. An infrared light source was used to observe the animals during the trials. For the sample phase of the NOR test, hamsters were placed along the center of a wall of the arena with two identical objects located on the opposite side in adjacent corners. Animals were allowed to freely explore the arena and objects for 5 min and then returned to their home cages. After a 24-h interval, one of the familiar objects from the sample phase and a different screened (novel) object were placed in the arena. Animals were allowed to explore for another 5 min during this test phase. Between each trial, the objects were removed, and both objects and the arena were cleaned with 70% ethanol, dried, and ventilated.

For the test phase, placement of the novel object was alternated from the left to right corner from trial to trial to prevent spatial biases in object exploration. Digital video recordings of the trials were scored by trained observers using a software program (XNote Stopwatch) to record the total time spent with each object. Exploratory behavior of the objects was defined as direct contact with the objects by the animal’s mouth, nose, or paws, or if the animal’s nose was within 1 cm of an object and its vibrissae were moving [9], [10]. Any indirect or accidental contact with the objects was not included in the scoring. Interobserver reliability was >95%. Preference for the novel object is expressed as a discrimination index (DI; DI  =  (time with novel object – time with familiar object)/total exploration time of both objects×100). Positive DIs indicate a preference for the novel object, whereas a value of zero indicates no preference.

### Drug Treatment

For each 10-day block of injections, PTZ (Sigma) was dissolved in saline and aliquoted into 5 vials on the first day, and again on the fifth day, of the injections and frozen (−20°C) until needed. Vials were defrosted on alternate days and refrigerated when not in use. PTZ (0.3 or 1.0 mg/kg, i.p.) or the vehicle (VEH) solution was injected daily for 10 consecutive days at 2 h before darkness (ZT14) in the animal room. An additional set of control animals (CON) was never injected or handled.

### Induction of Circadian Arrhythmia

Equal numbers of males and females were used in all groups and were 2–4 months of age at the start of the experiment. Hamsters were separated and housed singly in the same photoperiod as the colony room (LD 16∶8, lights on at 0200 PST). Rhythms were eliminated using a disruptive phase-shift (DPS) protocol as follows. Fourteen days after being housed singly, lights in the activity recording chambers were turned on for 2 h beginning 5 h after lights-off (i.e., a 2-h light pulse from ZT21–23). On the next day, the LD cycle was phase delayed by 3 h so that dark onset occurred 3 h later than on the previous night (lights on at 0500 PST). Animals remained in the 16∶8 LD cycle thereafter and locomotor activity was continuously recorded.

Activity data of animals that appeared arrhythmic based on visual inspection of their actograms were evaluated for circadian periodicity by Lomb-Scargleperiodogram analysis (ClockLab, Actimetrics, Evanston, IL) on 14-day blocks of data for each animal immediately prior to behavioral testing. Peaks in the periodogram were deemed statistically significant if they exceeded the 99.5% confidence interval limit. Animals were considered arrhythmic if there were no significant peaks in the periodogram in the circadian range, activity was distributed throughout the LD cycle, and if daily rhythm onsets and offsets could not be identified visually.Animals with confirmed loss of circadian locomotor rhythms (i.e., arrhythmic, ARR) at 4 weeks after the light treatment were randomly assigned to their experimental groups, along with age- and gender-matched controls from the hamster colony (i.e., entrained, ENT).

### Data Analysis

Performance on the NOR and SA tests was determined by a one-sample t-test to determine whether scores were statistically different from random chance performance (i.e., DI = 0 for NOR; alternations (%)  = 50 for SA) as recommended [44], [45]. Quantifying performance in this way for NOR is necessary because the magnitude of the novel object discrimination index is not an accurate reflection of memory strength in the NOR test [45]. Statistical evaluation of NOR and SA performance were based on specific hypotheses (i.e., planned comparisons) about the effects of arrhythmia and PTZ on memory and therefore did not require a corrected level of significance for multiple comparisons [44]–[46]. A score of positional bias was created to check for gender differences in left-right biases in the NOR arena or in the T-maze arms. Positional bias was calculated as: time on the right/(time on the left + time on the right) ×100, so that a score that is significantly <50% indicates a left bias, and >50% indicates a right bias. Changes in the number of arm entries and exploration time were evaluated by one- or two-way ANOVA (group×time of day) depending on the number of conditions being tested. The effects of PTZ on the number of arm entries and total exploration time were evaluated by two-way ANOVA with repeated measures for time. Data are presented as mean ± SEM.

## Results

### Sex Differences

Concerns have been raised recently regarding the bias towards the exclusive use of male animals in neuroscience research [47]–[50]. Given the well-documented gender differences in many neurological disorders [51]–[53], the inclusion of female animals in basic research is a worthwhile goal. We addressed this issue in the present study in two ways. First, we performed a study aimed at detecting sex differences in SA and NOR and, second, we balanced all subsequent experimental groups by sex and analyzed the data for any differences.

Table 1 presents the results from the sex differences experiment. There were no significant sex differences in performance on NOR or SA or in exploration time during the NOR test. Neither males nor females exhibited a significant positional bias in the NOR arena or in the T-maze (one sample t-test compared to 50%, P>0.05), and there were no sex differences in this measure (Table 1). Males did, however, make 14% more arm entries in the T-maze during SA than did females (see Table 1), but it was not associated with a significant increase in test performance.

**Table 1:** Sex differences in spontaneous alternation and novel object recognition performance.

	Males	Females	*t*-value	*P*
**Spontaneous Alternation**				
Alternation (%)	68.7 γ 2.8	67.0 γ 2.7	0.43	0.67
Arm Entries (#)	10.6 γ 0.6	9.1 γ 0.5	2.02	0.05
Positional Bias (%)	53 γ 2.1	58 γ 2.2	1.40	0.17
Sample Size	29	30		
**Novel Object Recognition**				
Discrimination Index	30.3 γ 3.3	27.4 γ 5.5	0.46	0.65
Exploration Time Sample Phase (sec)	15.5 γ1.7	14.1 γ 0.6	0.78	0.44
Exploration Time Test Phase (sec)	14.1 γ 1.0	15.8 γ 1.7	0.90	0.37
Positional Bias (%)	47 γ 2.0	51 γ 2.9	0.92	0.36
Sample Size	28	28		

### Elimination of CircadianLocomotor Activity Rhythms

**Figure 1:**
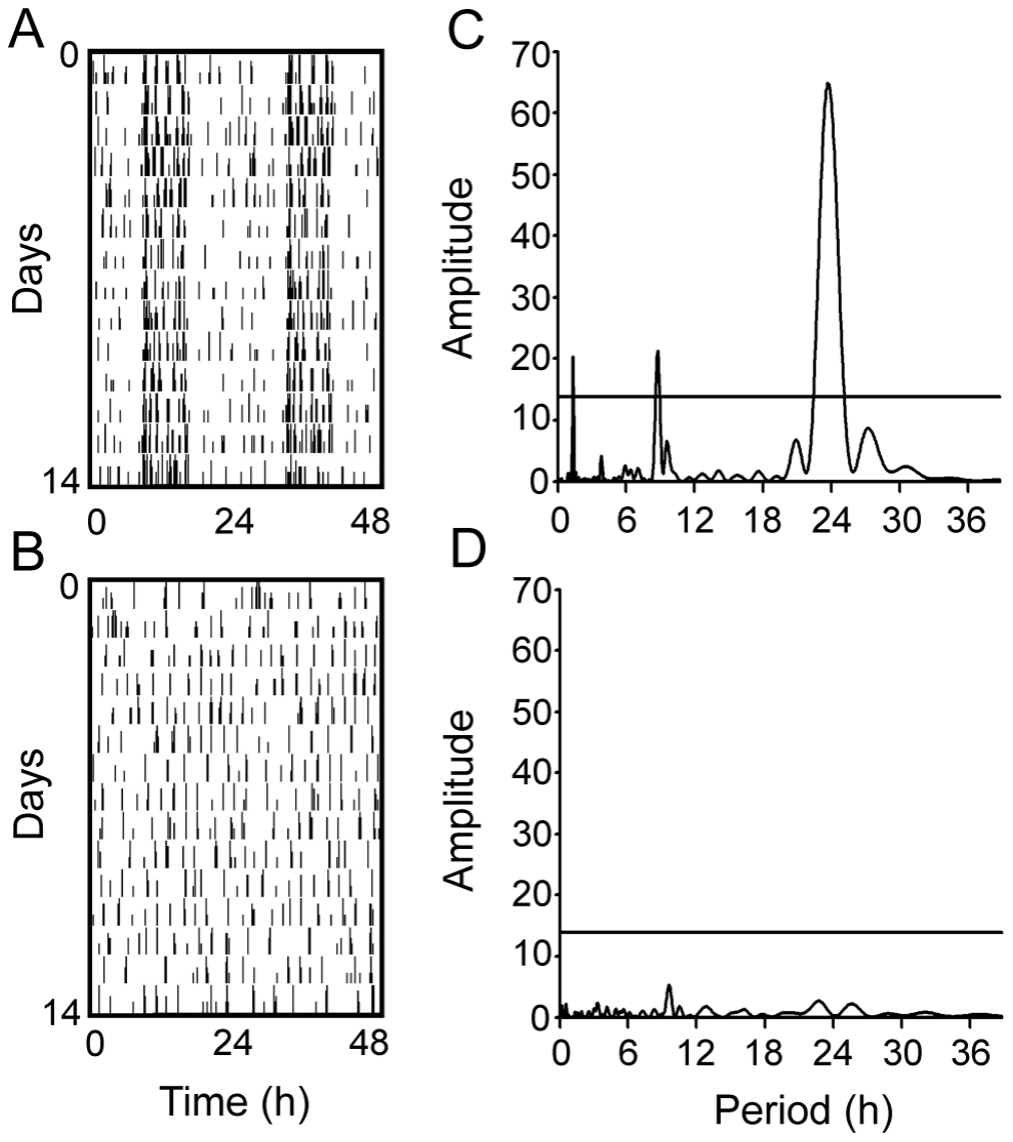
Representative actograms of a hamster exposed to the DPS protocol. (A) Locomotor activity before the DPS protocol is plotted horizontally in 10-min bins over 24 h and then double-plotted to facilitate visualization of the rhythms. (B) Activity four weeks after the DPS protocol. Successive days are plotted vertically beginning at day 0. (C) Plots of Lomb-Scargle time series analysis indicate significant periodicity in the circadian range when this animal was entrained. (D) Time series analysis confirmed that the DPS protocol eliminated circadian timing entirely. Peaks above the horizontal line indicate statistically significant periodicity (P<0.05).

The DPS protocol eliminated rhythms in 65% of hamsters within a few days after the light treatment (Fig. 1). None of the arrhythmic animals exhibited significant periodicity in the circadian range (Fig. 1). Their locomotor activity was distributed randomly across the light and dark phases of the LD cycle, whereas activity was mainly confined to the nighttime in control animals (Fig. 1). Of the remaining animals, 15% reentrained and the remainder had free running rhythms that failed to reentrain to the LD cycle as previously reported [33].

### Experiment 1. Effects of Circadian Arrhythmia and PTZ on Memory

A prior study showed that PTZ could rescue short-term performance on the NOR test when the interval between sample and testing phases was 60 min [36]. The experiment reported here evaluated whether PTZ could also rescue long-term (24 h) object recognition memory and spatial working memory. Animals were tested prior to PTZ treatment (Baseline) between ZT11-15 (i.e., late afternoon). The sample and test phases of NOR were separated by 24 h. Animals were allowed to remain undisturbed in their home cages for 3 days and then tested for SA from ZT11-15. Beginning 4 days after baseline testing was completed, arrhythmic animals were injected with PTZ or VEH daily for 10 consecutive days. The same groups of hamsters were evaluated in both tests on the day after the final injection (Day 1), and again 30 days later.

**Figure 2:**
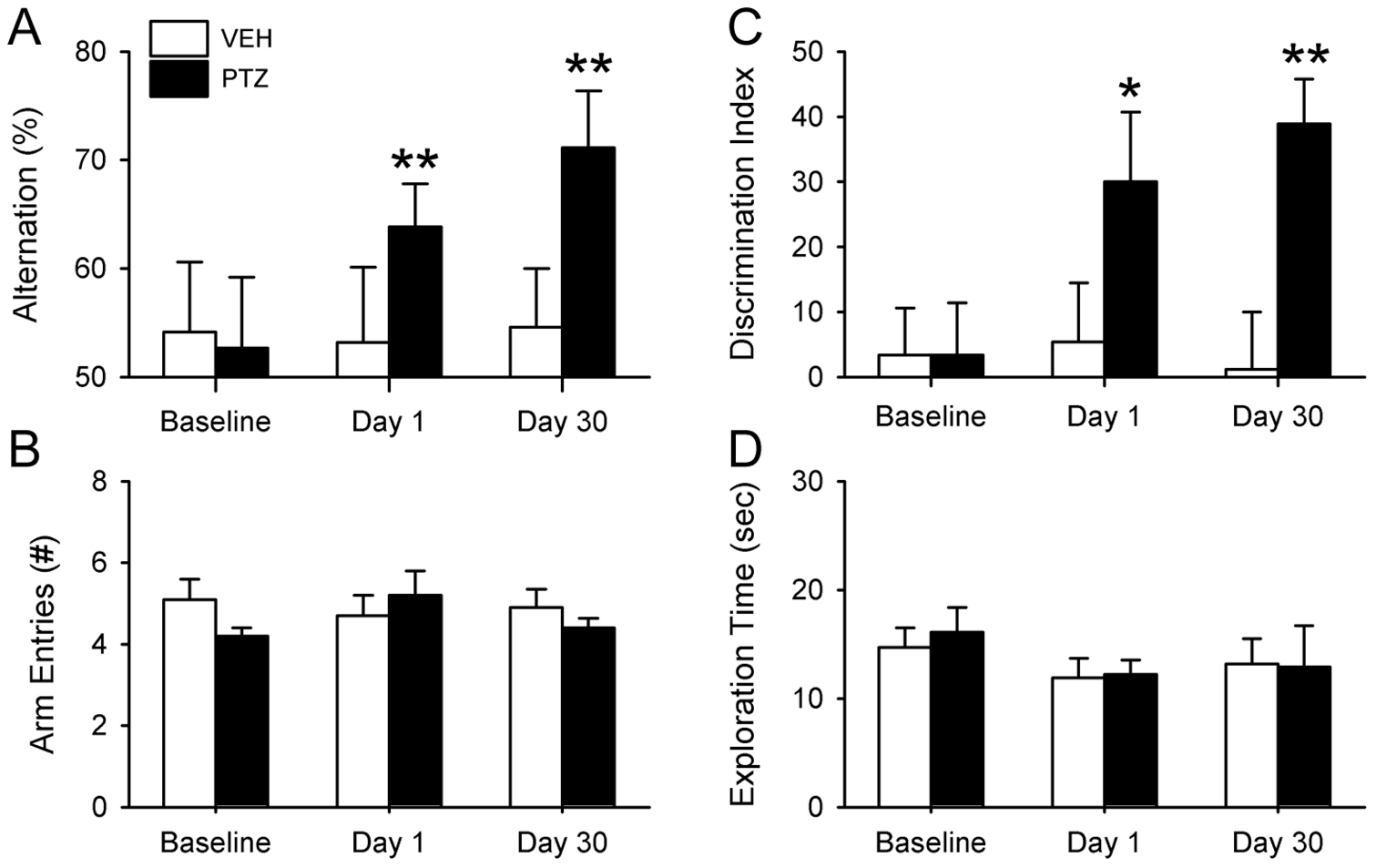
PTZ rescued memory in arrhythmic hamsters. Two groups of hamsters (PTZ, VEH) were tested prior to drug treatment (baseline) and at days 1 and 30 after treatment. (A) None of the ARR hamsters given PTZ or the VEH performed better than chance in the SA prior to PTZ treatment (baseline). PTZ restored test performance to normal levels and the effects lasted for at least 30 days after drug treatment. (B) PTZ had no effect on arm entries during the SA test. (C) PTZ restored long-term memory for object recognition in ARR hamsters. (D) Exploration time during the sample phase of NOR was not affected by PTZ. Test phase exploration time did not change over time and was not affected by PTZ (data not illustrated). VEH-treated ARR animals failed at both tests at all time points (P>0.05). * P<0.05, ** P<0.01 compared to random chance performance.

PTZ treatment did not restore circadian rhythms. Hamsters that were arrhythmic before PTZ treatment remained so after the injections and throughout the remainder of the study (data not shown). None of the hamsters performed better than chance in the SA and NOR tests prior to receiving drug (Fig. 2A, C). PTZ produced a marked improvement among arrhythmic (ARR) animals in SA and NOR performance that lasted at least 30 days after the drug regimen was stopped (Fig. 2). PTZ treatment improved SA behavior on days 1 and 30 (one-sample t-test, n = 10, P<0.01), whereas animals given VEH continued to make random arm choices in the T-maze at all three time points (P>0.05, n = 9; Fig. 2A). A similar pattern was found for NOR where the discrimination index improved significantly in PTZ-treated animals on days 1 and 30 (one-sample t-test, * P<0.05, ** P<0.01; Fig. 2C). Hamsters given the VEH solution failed to discriminate novel from familiar objects at any time point (P>0.05).

PTZ had no effect on the number of T-maze arm entries or on total object exploration time (Fig 2) at any time point. Each of these parameters was evaluated by two-way ANOVA (group: control, arrhythmic×trial: baseline, day1, day 30). For SA, there were no significant differences in the number of arm entries in the T-maze within or between groups across trials (P>0.05; two-way ANOVA, drug×trial; Fig. 2C). For NOR, sample and test phases were evaluated separately; there were no significant differences across trials among PTZ- or VEH-treated animals in exploration times for either phase at any time point (P>0.05; Fig. 2D).

### Experiment 2. Dose Effects of PTZ on Test Performance

This experiment was aimed at finding the minimally effective dose of PTZ and to see if that dose was different from the minimal PTZ dose that improved memory in Ts65Dn (Down syndrome) mice. PTZ rescues memory in Ts65Dn mice at a dose as low as 0.3 mg/kg [54]. We therefore compared a known effective dose (1.0 mg/kg) to the effects of 0.3 mg/kg. Animals were tested for NOR and then for SA three days later from ZT11-15.

**Figure 3:**
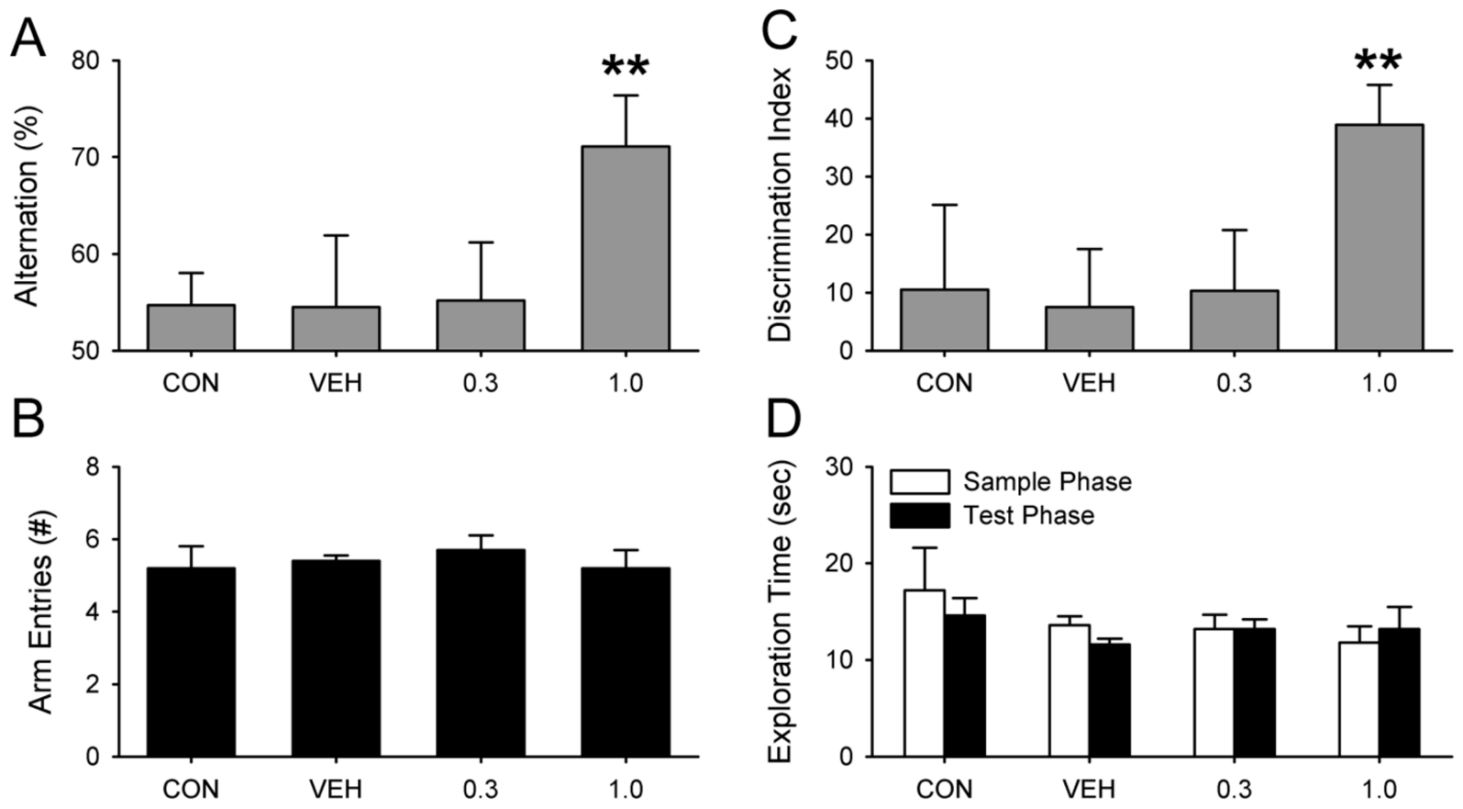
Dose response for PTZ in arrhythmic hamsters. (A) PTZ restored SA behavior when administered at 1.0 mg/kg (** P<0.01), but had no effect at 0.3 mg/kg (P>0.05). (B) PTZ dose had no effect on arm entries during SA (P>0.05). (C) PTZ was effective in restoring NOR only at the higher dose. Neither CON nor VEH animals performed better than chance (P>0.05). (D) PTZ had no effect on exploration times in NOR (P>0.05).

PTZ restored performance on the SA and NOR tests to normal levels in ARR hamsters treated with 1.0 mg/kg PTZ (n = 10), but had no effect when the dose was reduced to 0.3 mg/kg (n = 10; P>0.05; Fig. 2A, C). As in experiment 1, PTZ had no effect on the number of arm entries in the T-maze (one-way ANOVA) nor on the amount of exploration time in the NOR test in any of the four groups (two-way ANOVA with repeated measures for phase; P>0.05; Fig. 3B, D). There were also no differences in the time spent with both objects during either phase of the NOR test (P>0.05; Fig. 3D). ARR hamsters that were not injected (CON; n = 10) or given the vehicle solution (VEH; n = 10) failed at both tests (Fig. 3A, C).

The data from ARR animals injected with PTZ (1.0 mg/kg) were pooled from experiments 1 and 2 in an attempt to see if PTZ preferentially rescued memory in males (n = 10) or females (n = 10), but t-tests did not reveal any significant differences in discrimination index, alternation %, exploration times (sample and test phases), or in the number of arm entries (P>0.05 for each measure).

### Experiment 3. Circadian Modulation of Spontaneous Alternation

The goal of this experiment was to assess whether the circadian system modulates SA performance across the day/night cycle as it does for NOR [36]. This experiment was also essential because time of day effects had to be established before we could test whether PTZ affects cognition in ENT animals. SA testing was done with 12 different sets of animals (6 control and 6 arrhythmic) at six different times of day, equally spaced across the day and night.

**Figure 4:**
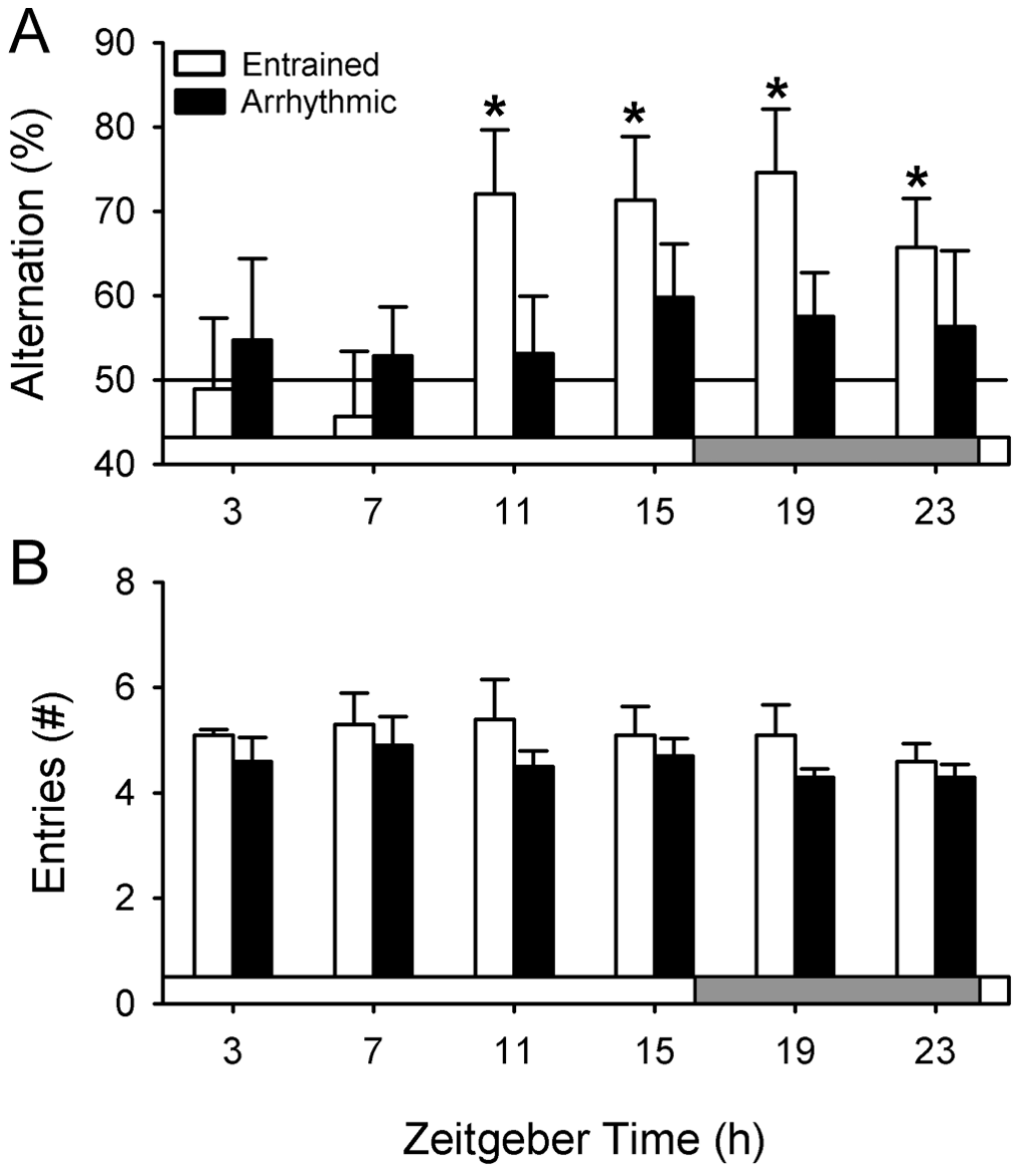
Entrained hamsters exhibit a daily rhythm in spontaneous alternation, but not in exploratory behavior. (A) ENT animals performed well on the SA test late in the day and at night, but not early in the day. By contrast, ARR hamsters could not perform this test successfully at any time of day. Gray bar indicates the time of the 8-h dark phase. * P<0.05 compared to random arm choices (i.e., 50%). (B) Exploratory behavior (i.e., number of arm entries) did not change across the day and did not differ among ENT and ARR hamsters at any single time point (P>0.05).

There was a robust daily rhythm in performance on the SA test among ENT animals (n = 9–10 per group at each time point; Fig. 4A). ENT hamsters exhibited significant alternation rates late in the afternoon and at night (one-sample t-test comparing performance to random chance (i.e., 50%; P<0.05), but not early in the light period (one-sample t-test, P>0.05). By contrast, ARR hamsters could not perform the SA test at any time of day or night, as their alternation scores did not exceed chance levels (n = 9–10 per group; one-sample t-test, P>0.05; Fig. 4A).

The number of arm entries made in the T-maze during the SA test was compared among ENT and ARR hamsters across the six time points by two-way ANOVA. There was no effect for time of day (P>0.05; Fig. 4B). However, ARR hamsters made significantly fewer arm entries than did ENT animals (F_(1,104)_ = 5.51, P<0.05; 4.6±0.2 vs. 5.2±0.2 entries for ARR and ENT animals, respectively). There were no significant interactions between time of day and circadian rhythm condition. Data from ZT11-23 were pooled among ENT animals to test for sex differences. There were no significant differences between males (n = 19) and females (n = 20) in alternation rates or in the number of arm entries (t-tests, P>0.05 for both measures).

### Experiment 4. PTZ Effects on Memory of Entrained Animals

**Figure 5:**
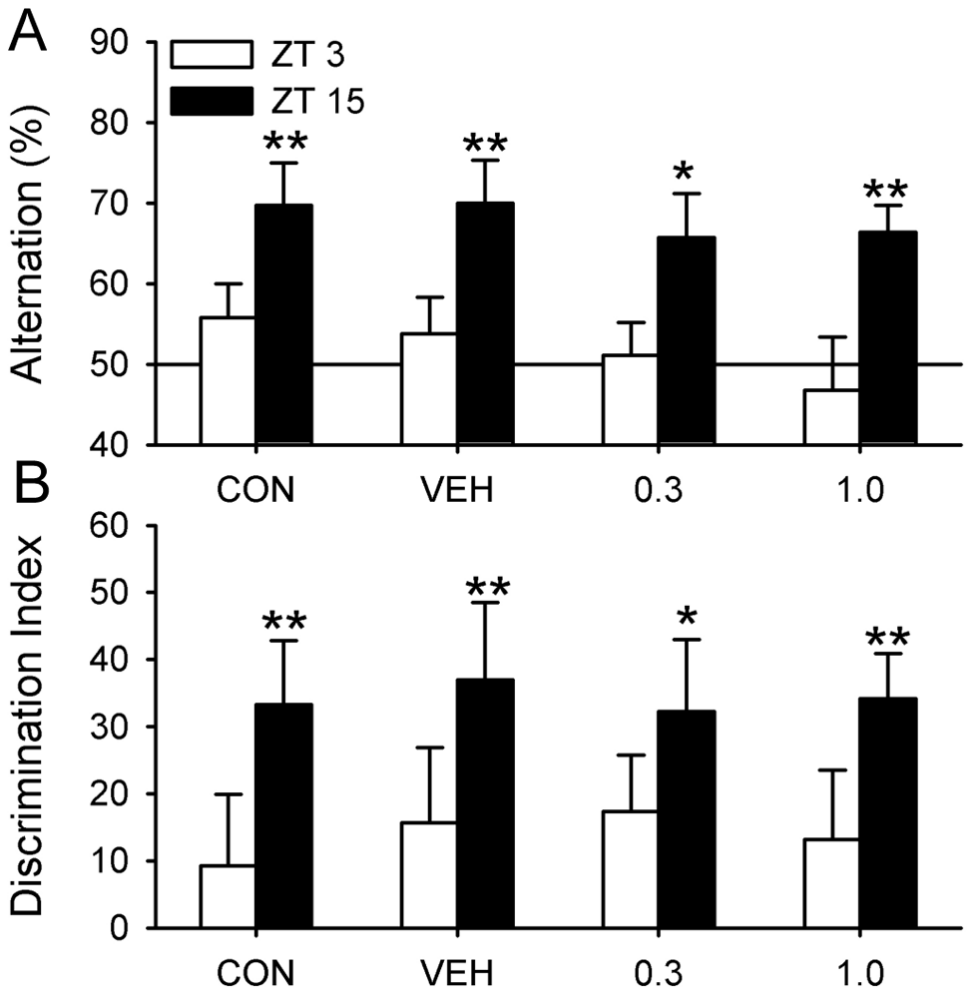
PTZ did not improve memory of entrained animals in either test. Separate groups of animals were tested on either SA or NOR. All animals were tested at ZT3 and re-tested 3 days later at ZT15. (A) All of the ENT animals alternated in the T-maze at rates significantly above chance levels when tested in the late afternoon (ZT15), but performed at chance levels early in the morning (ZT3; n = 9–10 per group; P>0.05). (B) Hamsters readily discriminated between novel and familiar objects in the NOR test at ZT15, but not at ZT3 (n = 7–8 per group; P>0.05). PTZ did not improve test performance at ZT3 or at ZT15. * P<0.05, ** P<0.01.

The recovery of memory in PTZ-treated ARR hamsters raised the possibility that PTZ might improve memory in ENT hamsters during the early morning when they normally fail at the SA and NOR tests. We therefore administered PTZ to ENT animals using the same 10-day injection regimen that was used with ARR hamsters. All injections were given at ZT14 (2 hours before lights-off). Animals were assessed 30 days after the injections because improvements in memory are greater on day 30 than they are on day 1 after injections in ARR hamsters (Fig. 2), and because PTZ effects last for at least 60 days in entrained mice [55]. Unlike ARR animals, ENT hamsters were tested on either the SA or NOR, but not both, so that an individual animal was not tested more than once in the morning. This was done to prevent any possibility that the animals would exhibit increased arousal in anticipation of being handled at the time of day when they are normally sleeping. Each animal was tested at ZT3 followed by testing at ZT15 three days later. As expected from prior work (experiment 3, [36]), animals tested in the afternoon (ZT15) performed significantly better than chance on both tests, whereas animals tested in the morning (ZT3) did not (P>0.05; Fig. 5). PTZ did not improve SA or NOR performance at either ZT3 or at ZT15 (Fig. 5A, B).

**Figure 6:**
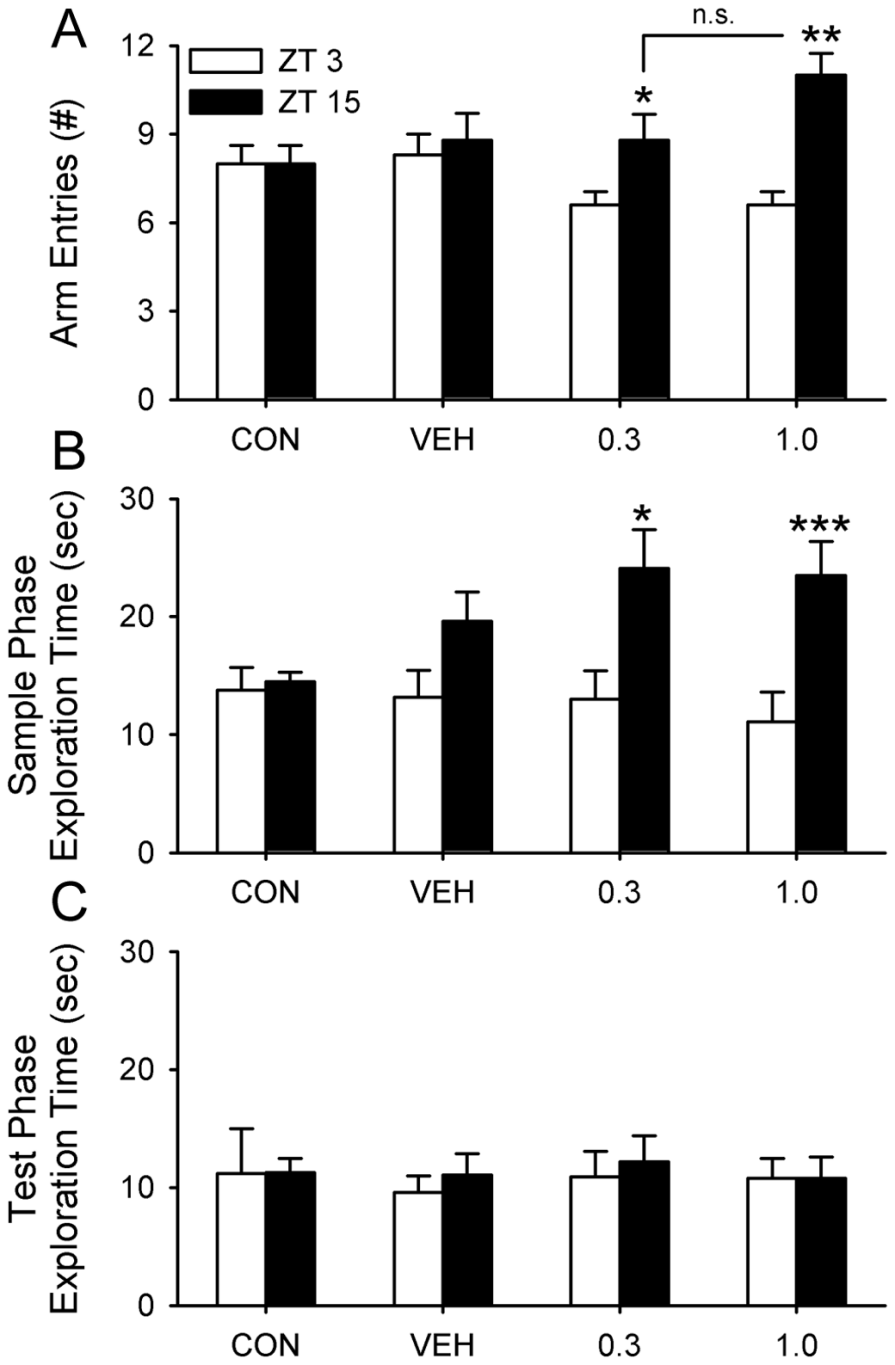
PTZ increased exploratory behavior in entrained animals during memory tests. Arm entries and exploration times for animals from figure 5. (A) The number of arm entries made during SA at both doses of PTZ were higher in the afternoon at ZT15 compared to the early morning at ZT3. This effect appeared to be due to a decrease in arm entries at ZT3, but this apparent reduction was not statistically significant (see text for analysis). (B) A similar effect of PTZ was found for time spent exploring objects during the sample phase of NOR. (C) PTZ had no effect on exploration time 24 h later during the test phase. * P<0.05, ** P<0.01, *** P<0.001 for ZT3 vs. ZT15 comparisons;n.s.  =  nonsignificant difference.

There were significant effects of PTZ on exploratory behavior in both tests (Fig. 6). In regards to the number of arm entries made during SA, a two-way ANOVA (group×time of day; repeated measures for time of day) revealed a significant effect for time of day (F_(3, 72_ = 10.91, P<0.001), but not for treatment group (P>0.05; Fig. 6A). There was, however, a significant interaction between these two variables (F_(3,72)_ = 4.91, P<0.01), so pairwise t-tests (Tukey’s post-hoc correction applied) were performed for time of day. Hamsters treated with PTZ made significantly more arm entries at ZT15 compared to ZT3 at both doses (0.3 mg/kg, P<0.05; 1.0 mg/kg, P<0.001; Fig. 6A).

A similar analysis was done for exploration time during the sample phase of the NOR. A two-way ANOVA revealed a significant effect for time of day (F_(3, 58)_ = 20.83, P<0.001), but not for group (P>0.05; Fig. 6B). Pairwise t-tests for time of day showed that hamsters treated with PTZ spent significantly more time exploring the objects during the sample phase of the NOR at ZT15 compared to ZT3 (0.3 mg/kg, P<0.05; 1.0 mg/kg, P<0.001; Fig. 6B). There were no significant differences among groups or time of day for time spent exploring the objects during the test phase (P>0.05; Fig. 6C).

**Figure 7:**
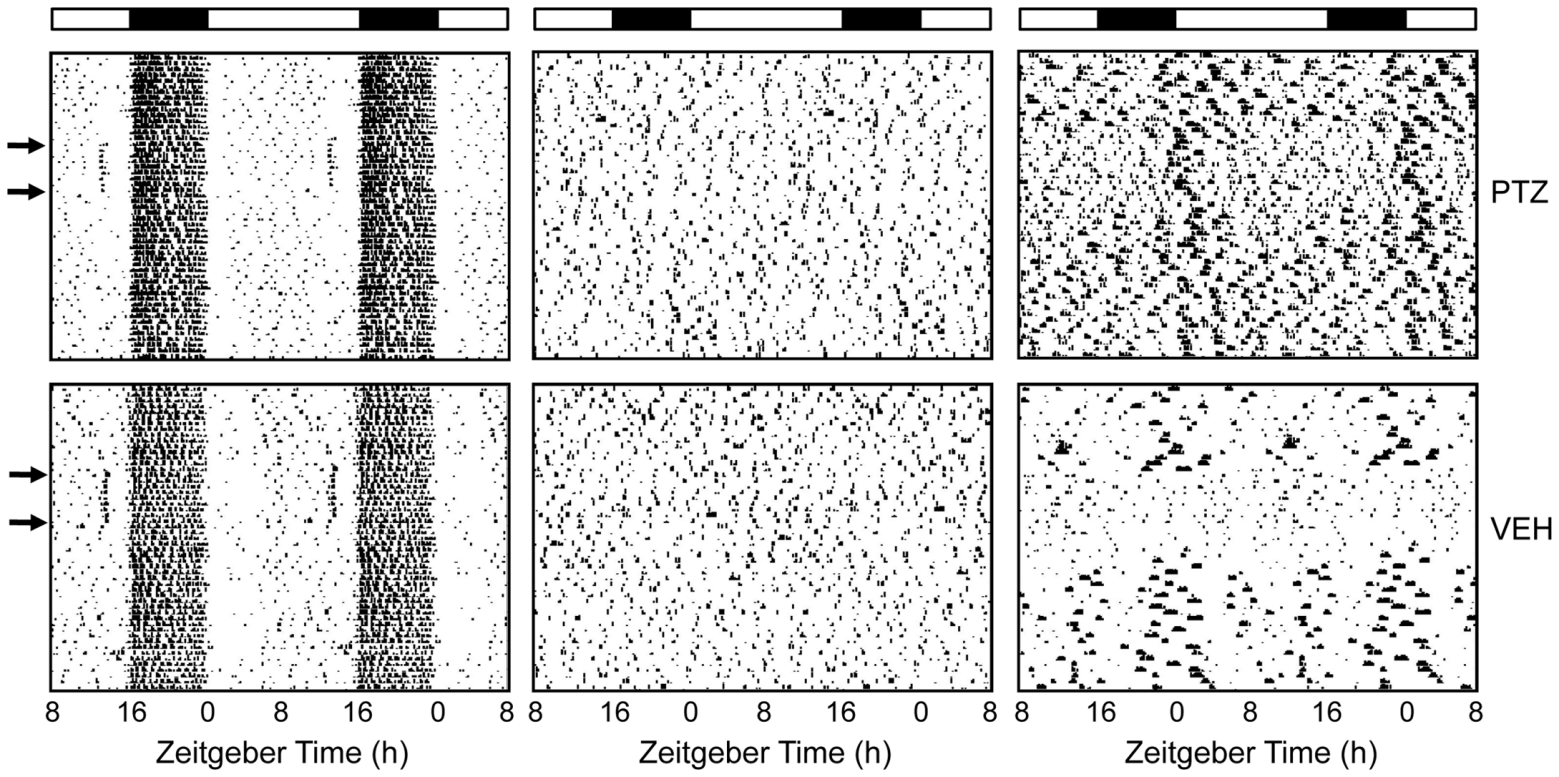
Representative actograms of entrained and arrhythmic hamsters showing the effects of drug injections on locomotor activity. Brief bursts of locomotor activity were exhibited by ENT (left panels), but not by ARR (middle panels) animals, around the time of drug injections. Locomotor activity patterns were altered by the injection regimen in only two ARR animals (right panels). Those two animals decreased the amount of daily activity until after the end of the injections. Arrows indicated the first and last day of injections that were given at ZT14 each day. Light and dark bars represent the daily illumination cycle.

PTZ had no effect on the amount of locomotor activity of ENT or ARR animals (Fig. 7). Mean daily activity in the animal’s home cages was compared during three 10-day periods before, during, and after injections of PTZ or VEH. Activity levels did not change significantly during these times (one-way ANOVA with repeated measures for PTZ and VEH groups; P>0.05). Activity levels were not compared between groups. Both PTZ and VEH injections produced a brief burst of locomotor activity after each daily injection in ENT animals (Fig. 7, left panels). Because this effect is typical of drug injection studies, we expected to observe the same effect in the ARR hamsters, but there was no evidence of it (Fig. 7, middle panels). Two ARR animals did, however, alter their activity patterns in response to PTZ and VEH injections. Both of these animals reduced their daily activity at the start of the injections, and resumed pre-injection activity levels after the injections were terminated (Fig. 7, right panels).

## Discussion

Siberian hamsters have a labile circadian pacemaker that can be made permanently arrhythmic by light [32], [33]. The primary advantage of this model of circadian arrhythmia is that it eliminates rhythms while leaving the animals genetically and neurologically intact. A secondary advantage is that Siberian hamsters rendered arrhythmic by this method do not exhibit changes in their sleep architecture or in their homeostatic responses to sleep deprivation, and thus provide an opportunity to dissociate the effects of sleep from those of circadian timing on declarative memory. The relative amounts of rapid eyemovement (REM) sleep, non-REM sleep, and slowwave activityexpressed by Siberian hamsters at times ofday when they can (e.g., ZT11) or cannot (e.g., ZT7) perform the NOR and SA tests are indistinguishable from one another [35], [36]. This situation is similar to that of humans where circadian effects on cognition have been shown to be distinct from those of prior sleep history [56].

Using this model we showed that ARR hamsters have significant memory deficits that are rescued by chronic PTZ administration [36]. The NOR and SA tests require normally functioning hippocampal or septal-hippocampal circuits. Thus, memory impairments caused by circadian arrhythmia might derive from changes in the excitability of these circuits or conceivably in others that comprise the medial temporal lobe. Although ENT hamsters succeeded at the SA and NOR tests in the afternoon and at night, they failed early in the day [36]. Test performance in ENT animals was completely unaffected by PTZ, but the drug did increase their exploratory activity. This shows that a low dose of PTZ (1.0 mg/kg daily for 10 days), while having no effect on cognition, was sufficient to produce changes in brain function in normal animals for at least 30 days after the treatment. All of these effects were observed in both males and females. Given the known sex differences in memory performance among other rodent species [57]–[58], we conclude that the lack of sex differences among Siberian hamsters indicates that any real differences were too subtle to be detected by the tests we used, or that sex differences in spatial and recognition memory are not very pronounced in this species.

The poor performance of ARR animals seems to reflect a true memory deficit. Exploratory behavior, as defined by the number of arm entries in the T-maze for SA, or by the time spent exploring objects in NOR, was constant across the day and night in both ENT and ARR hamsters, whereas test performance was cyclic in control animals [36]. This pattern suggests that animals were equally motivated to attend to the memory tests at all times of day whether or not they could actually perform the test. Although the number of arm entries and the time spent exploring objects are a crude measure of motivational state, this pattern is consistent with the behavior of ARR animals in the short-term NORtest where exploration time was also unaffected by time of day in both ENT and ARR hamsters [36]. We previously reported that performance of the ARR hamsters in the NOR test was inversely related to the delay between sample and test phases [36]. Such delay-dependent performance is indicative of an impairment in memory rather than a deficit in motivation, attention, or perception, which are factors that are independent of the delay interval [59]. Insofar as one can infer motivational state solely by behavior, motivation in both the NOR and SA tests is constant and not under circadian control.

ARR animals that received PTZ were still arrhythmic after drug treatment, but performed as well as ENT animals did at ZT15, which suggests that hippocampal function was normal in these hamsters. Several decades of research have demonstrated that the hippocampal system and adjoining circuitry in the septum, entorhinal cortex, and prefrontal cortex make specific contributions to SA behavior that are not matched by other areas of neocortex or by other areas within the limbic system such as the amygdala, bed nucleus of striaterminalis, or cingulum [60]. SA behavior is, thus, effectively a direct test of hippocampal system integrity. Another significant body of work has demonstrated the important roles of the perirhinal cortex and hippocampus in contextual object recognition memory [61]–[63]. PTZ is a non-competitive antagonist of the GABA_A_ receptor that binds within the channel pore to block the passage of chloride ions through the channel [64]. PTZ has a half-life of approximately 1–2 hours *in vivo* with high bioavailability throughout the brain and a history of clinical use, where it has been previously employed to treat various neurological and psychiatric disorders, and as a brainstem respiratory and vasomotor stimulant [65]–[67]. PTZ-induced kindling is routinely used as a seizure model to screen antiepileptic drugs [68], by injecting it every few days at very high doses (e.g., 20–80 mg/kg per injection) until it induces seizures that often originate in the medial temporal lobe, which encases the hippocampus [69].

Although we cannot presently say for sure where PTZ exerts its actions in the ARR hamster brain, the behavioral paradigms we used, combined with previous studies in rodents conducted with high or low doses of PTZ, suggest that the hippocampus is a very likely target.While GABA_A_ receptors are widely distributed throughout the brain, hippocampal circuits demonstrate more sensitivity to the actions of GABA_A_ antagonists than do other brain circuits [70]–[73], with selectivity of action seen even when high PTZ doses are employed to trigger seizure [70]–[73]. Low-dose PTZ regimens elicit beneficial changes to hippocampal synaptic plasticity, cell composition, and morphology in rats and mice, including Ts65Dn mice [55], [74], [75]. Moreover, a rich historical literature has chronicled how low-dose GABA_A_ antagonist treatments improve consolidation in tests of declarative memory that putatively involve the hippocampus (reviewed in [76]).

We previously proposed that an arrhythmic SCN could increase the relative amount of inhibition in the hippocampus via a pathway between the SCN, septum, and hippocampus [36]. In this model, noncircadian firing patterns of SCN neurons would produce a steady GABAergic output to the lateral/medial septum, which, in turn, would attenuate cholinergic signaling and interrupt normal daily oscillations of excitatory and inhibitory activity within the hippocampus. The present results suggest that PTZ improves memory by raising constitutive hippocampal excitability in a way that compensates for the loss of excitability arising from SCN-inhibition of septal activity (“therapeutic neuroadaptation”) [77], though they do not preclude the possibility that chronic PTZ treatment could also curtail SCN GABAergic output.

PTZ improved memory in ARR hamsters but did not augment cognition in ENT animals, even in the morning when ENT hamsters performed poorly in both SA and NOR tests [36]. Similar results have been reported for Ts65Dn (Down syndrome) mice and for a rat model of diencephalic amnesia where blocking GABA_A_ receptors with either systemic injections of PTZ or by directly injecting bicuculline into the medial septum restored SA behavior to normal levels in cognitively impaired animals, but had no effect in controls [55], [78]. The failure of PTZ to improve memory in control animals is an important observation because it supports the view that mnemonic deficits in these animal models and in ARR hamsters arise from specific abnormalities in declarative memory and are rescued by drug effects within these circuits, rather than to some nonspecific effects of the drug [55], [78], [79]. The over-inhibition model suggests that the doses used here were minimally sufficient to balance hippocampal excitability and improve memory without causing excessive excitation [77]. This is evidenced by the marked differences in efficacy between 0.3 and 1.0 mg/kg PTZ doses on memory in ARR hamsters. The observed increases in the amount of exploration time during the NOR sample phase and during SA in ENT animals confirms that these doses were sufficient to alter brain activity, but it also suggests that there are limits to the benefits of attenuating inhibition in normal animals. It is unlikely that higher doses would augment cognition given that the doses used here already had substantial effects on exploratory behavior. It is noteworthy that the increases in exploration among ENT hamsters were not due to PTZ-induced hyperactivity as PTZ did not increase the amount of locomotor activity in the home cages, nor did it increase the amount of activity in the NOR test phase. Exploration times were already higher for these animals during the initial exposure (sample phase) to the NOR arena than they were during the test phase, and PTZ may have exaggerated this effect.

PTZ treatment did not restore circadian locomotor rhythms in ARR hamsters nor did the drug alter locomotor activity in most of the animals. Nevertheless, we cannot rule out the possibility that daily PTZ injections initiated daily oscillations in non-SCN oscillators or in the hippocampus, which in turn might improve memory. Animals bearing complete SCN lesions continue to exhibit daily rhythms in conditioned place avoidance and conditioned place preference [80], [81], and are able to use time of day as a discrimination cue [82]. In the absence of the SCN, a food-entrainable oscillator might provide timing cues [82]. To sustain this explanation for the ARR hamsters, PTZ would have to stimulate oscillations in the brain without having an impact on locomotor activity rhythms, which does not seem likely given that GABA_A_receptors are spread throughout the nervous system. These oscillations would also have to last at least 30 days after the PTZ treatment, which is when the animals were tested, and these rhythms would have to entrain to the LD cycle. While PTZ-induced rhythms in the hippocampus could potentially explain our data, it is important to note that circadian rhythms in the hippocampus are not necessary for object recognition or spatial memory. SCN lesions eliminate hippocampal rhythms, but have no effect on NOR performance and do not impair escape latency in the Morris water maze, which is a well-documented measure of spatial memory [83]. Furthermore, ablation of the SCN accelerates acquisition of discrimination trial learning, suggesting that the SCN may play some role in learning time-of-day discrimination [82].

Many people experience the adverse effects of disrupted circadian timing such as memory deficits because of aging, shift-work, transmeridian travel, or disease. A direct role of the circadian system in memory disorders has only recently received broad recognition. The arrhythmic Siberian hamster model offers a novel perspective on the underlying mechanism of this important health issue, and points to the use of GABA antagonists to mitigate at least some of the cognitive problems associated with circadian arrhythmia.
