# Correction: Spatial Memory and Long-Term Object Recognition Are Impaired by Circadian Arrhythmia and Restored by the GABA_A_Antagonist Pentylenetetrazole

**DOI:** 10.1371/annotation/f9111e45-33ca-47d8-b6a5-be277c292fdc

**Published:** 2013-10-15

**Authors:** Norman F. Ruby, Fabian Fernandez, Alex Garrett, Jessy Klima, Pei Zhang, Robert Sapolsky, H. Craig Heller

In multiple sections of the article, the terms "*Phodopus*" and "*sungorus*" were incorrectly joined together. In addition, in Table 1, the sign "±" was incorrectly replaced by the sign "γ" at multiple points. 

